# Bipolar Disorder, Brain-Derived Neurotrophic Factor (BDNF) Val66Met Polymorphism and Brain Morphology

**DOI:** 10.1371/journal.pone.0038469

**Published:** 2012-07-30

**Authors:** Cheryl Ann Teh, Tih-Shih Lee, Margaratha Kuchibhatla, Allison Ashley-Koch, James MacFall, Ranga Krishnan, John Beyer

**Affiliations:** 1 Neuroscience and Behavioral Disorders Program, Duke-National University of Singapore Graduate School of Medicine, Singapore, Singapore; 2 Department of Psychiatry and Behavioral Sciences, Duke University Medical Centre, Durham, North Carolina, United States of America; 3 Department of Biostatistics and Bioinformatics, Duke University Medical Centre, Durham, North Carolina, United States of America; 4 Department of Medical Genetics, Duke University Medical Centre, Durham, North Carolina, United States of America; 5 Department of Radiology, Duke University Medical Centre, Durham North Carolina, United States of America; Hangzhou Normal University, China

## Abstract

In this study of the effect of bipolar status and presence of BDNF Val66Met polymorphism on differences in regional brain volumes, we hypothesized based on previous studies that 1) bipolar subjects will have smaller regional brain volumes than healthy controls; 2) BDNF Met66 allele carriers within the same population are likely to have smaller regional brain volumes as compared to Val66 homozygyotes. In our Caucasian sample of 166 bipolar subjects and 64 gender-matched healthy controls, we found significant decreases in total (p = 0.005) and regional gray matter volumes in bipolar patients compared to healthy controls, more pronounced in the inferior and posterior parts of the brain, together with a concomitant increase in total CSF (p = 0.012) particularly in the lateral ventricles (p = 0.023). However, there was no difference in white matter volumes noted by other studies. Furthermore we did not find significant differences in other brain regions that have been reported by other authors. Nor did we find a significant effect of BDNF on these measurements.

## Introduction

Bipolar disorder is a significant psychiatric condition that affects approximately 1–3% of the population [1]. The pathophysiology of this disease remains poorly understood, though a dysregulation of the anterior limbic network is thought to be involved. Neuroimaging research on this disease has focused on regional differences of specific cortical volumes, for example, attempting to evaluate network components by quantifying changes in both the gray and white matter components of the brain [2], [3]. Various neuroanatomical changes have been observed in structural MRI studies of bipolar patients compared to healthy subjects, most notably, increased ventricular volumes (total lateral, right lateral ventricle and third ventricle) [4] with corresponding regional grey matter reductions in various subcortical structures implicated in emotional processing, including the anterior cingulate and insula cortex [5], amygdala [6] and hippocampus [7].

There has been compelling evidence that genetic factors are important in determining susceptibility to bipolar disorder. A candidate gene encoding Brain-Derived Neurotrophic Factor (BDNF) has been implicated in bipolar disorder [8], [9] and other psychiatric conditions. BDNF potentiates release of dopamine in nucleus accumbens and is a key regulator of the mesolimbic dopamine pathway via activation of tyrosine kinase receptors, regulating identification of and response to emotionally salient environmental stimuli [10]. With regard to the pathogenesis of bipolar disorder, studies have shown a reduction in serum BDNF levels during acute manic and depressive episodes with recovery to levels similar to that of controls following treatment and remission to euthymic state [11], [12], [13], as well as decrease in BDNF levels with age and length of illness [12]. Of particular interest is a single nucleotide polymorphism (SNP) in chromosome 11p13 in the BDNF gene, found to exist in no other vertebrate species except humans [14]. A valine(G) to methionine (A) substitution in the 5′ proregion, specifically on nucleotide 196, codon 66, produces the SNP. This polymorphism appears to confer genetic susceptibility to a range of psychiatric disorders including anxiety disorders [15], [16] schizophrenia [17] and of particular relevance to this paper, mood disorders – both unipolar depression, in particular late life depression [18], [19], [20], and bipolar disorder [8], [21], [22]. It has been associated with the rapid cycling variant of bipolar disorder [22]. Some studies [8], [21] have found that the Val allele is over transmitted in bipolar disorder, a pattern also found in schizophrenia, while others [9] have reported no association between variation at the BDNF Val66Met locus and susceptibility to bipolar disorder.

In addition to influencing both susceptibility and clinical course of psychiatric disorders, the BDNF Val66Met polymorphism has also been found to impact brain morphology [23], [24], [25]. The presence of a Met66 substitution in neurons and neurosecretory cells leads to three trafficking defects: 1) decreased variant BDNF distribution into neuronal dendrites, 2) decreased variant BDNF targeting to secretory granules, and 3) subsequent impairment in regulated secretion [25], [26]. When expressed together in the same cell, Met66 allele alters the intracellular trafficking of “wild type” BDNF (Val allele) through the formation of heterodimers that are less efficiently sorted into the secretory pathway [27], with an overall effect of decreased secretion of BDNF at the synapse in the presence of Met66 allele. Given that BDNF plays an important role in neurogenesis, neuronal protection, cell survival and synaptogenesis in the brain, it follows that Met66 allele carriers should have smaller brain volumes – both total and regional – than Val/Val homozygotes. Numerous studies have demonstrated regional differences in the brains of individuals carrying BDNF val66 or met66 alleles, specifically in gray matter of the prefrontal cortex [27] and hippocampal volumes [27], [28], [29] in healthy populations as well as bipolar populations [7], [30].

The major focus of structural neuroimaging so far has been to define regional changes either through region of interest, voxel-based mapping or density studies. In this study, we utilized a regional parcellation technique that enabled us to examine volumetric regions of gray and white matter and cerebrospinal fluid, identical to what was done in [2] with the inclusion of additional regions and structures of the brain previously not analyzed. In addition, the average age of this population was younger than in previous studies we conducted [2], [31], [32], [33] to reduce the potential confounding effect of normal aging on brain volumes, given that neurotrophins, including BDNF, have been found to play a critical role in normal aging [34].

### Hypothesis

On the basis of previous work done by ourselves and others in psychiatric and healthy populations [2], [4], [31], we predicted that bipolar subjects will have smaller regional brain volumes than healthy controls, in particular inferior frontal gray matter regions, with a compensatory increase in the volume of ventricular and non-ventricular cerebrospinal fluid. Within the same study population, BDNF Met66 allele carriers (controlled for healthy vs. bipolar status) are likely to have smaller brain volumes as compared to Val66 homozygotes, in particular that of gray matter regions of the prefrontal cortex.

## Methods

### Sample & Clinical Evaluation

Subjects with bipolar disorder were recruited from outpatient clinics at Duke University Medical Center in Durham, NC. Comparison subjects were recruited through community advertisements. The study was approved by the Duke University Health System Institutional Review Board. After complete description of the study to the subjects, written informed consent was obtained.

All subjects completed the SCID (Structured Clinical Interview for DSM-IV) to assess for psychiatric diagnoses. Bipolar subjects met DSM-IV criteria [35] for Bipolar I disorder. Comparison subjects did not meet diagnostic criteria for any psychiatric diagnosis. All subjects had to be 18 years or older to participate. Exclusion criteria included meeting DSM-IV diagnostic criteria for another Axis I psychiatric illnesses, a current manic episode, and active substance abuse or dependence. Subjects who were acutely manic were excluded as it would be difficult to ensure that they stayed still in the MRI scanner. Exclusion criteria further included any neurological illness, evidence of cognitive impairment (as suggested by a score of ≤23 on the Mini Mental Status Exam (MMSE) [36] or contraindications to MRI.

Demographic data were gathered through the clinical evaluation, including a list of current medications and self-report of the number of previous mood episodes. When subjects could not provide documentation of their medication regimen, it was confirmed with the prescribing physician. Current depressive symptoms were assessed using the Center for Epidemiological Studies – Depression (CES-D) scale [37].

As we had previously found a racial difference in frequency of the Val66Met polymorphism [18], we restricted this analysis to Caucasian American subjects only. Demographics of our study population are presented in Table 1.

**Table 1:** Group Differences by Bipolar Status and BDNF Val66Met Genotype.

	Bipolar	Controls	Total n	P-value
**N**	166	64	230	
**Female** (**%**)	110 (66.3)	50 (78.1)	160 (69.6)	p = 0.0798
**Mean Age**(**SD**)**/years**	44.36 (13.70)	49.41 (16.77)		p = 0.0343
**BDNF Genotype**	**Met allele carriers**	**Val homozygotes**		
**N**	92	138	230	
**Female** (**%**)	62 (67.4)	98 (71.0)		p = 0.5585
**Mean Age** (**SD**)**/years**	47.55 (14.38)	44.59 (14.94)		p = 0.1385

### Genotyping

Fresh blood samples were obtained from all participants and DNA extracted and stored according to methods and quality checks as previously reported [38]. An aliquot of DNA was used for genotyping of the BDNF Val66Met polymorphism. DNA samples were placed in 96-well plates together with no-template controls and four sample duplicates in an asymmetric pattern to avoid unintended plate-switching. DNA was polymerase chain reaction-amplified applying a Taqman by-design assay (Applied Biosystems) that recognized the single nucleotide polymorphism, which defines the Val66Met polymorphism (rs6265). The samples were analyzed using an ABI7900 DNA analyzer (Applied Biosystems) and the genotypes determined with the SDS software package (Applied Biosystems). Greater than 95% genotyping efficiency was required before data was submitted for further analysis.

### Magnetic Resonance Imaging (MRI) Acquisition and Analysis

1.5T MRI acquisition of axial images was performed on a whole-body system (SIGNA, GE Medical Systems, Milwaukee, WI) using the standard head (volumetric) radiofrequency coil. The scanner alignment light was used to adjust the head tilt and rotation so that the axial plane lights passed across the cantomeatal line and then sagittal lights were aligned with centre of the nose. A rapid sagittal localizer scan confirmed the alignment.

An axial dual-echo fast spin-echo acquisition was obtained. The images were acquired in two separate acquisitions with a 3 mm gap between sections. The second acquisition was offset by 3 mm from the first to create contiguous sections. Images were processed in DUMC’s Neuropsychiatric Imaging Research Laboratory (NIRL) on SUN workstations. A semi automated method was used to convert image intensity to segmented tissue types, making use of the multiple MR contrasts available to identify different tissue classifications through a “seeding” process, in which a trained analyst manually selects pixels in each tissue type to be identified (gray matter, white matter, cerebrospinal fluid, lesions or background). The seeding protocol identifies the range of signal intensities that characterize each tissue type.

Subdivision of the cerebrum into component parcellation regions was performed using the GRID program which was developed by NIRL. Each scan was re-aligned to a standard orientation, including making the anterior commissure–posterior commissure (AC–PC) plane horizontal. Following this, the planes were defined. First, a mid-sagittal plane was used to divide the left and right cerebral hemispheres. Then, an axial plane was created along the AC–PC plane, dividing superior regions from inferior. Next, coronal planes were created perpendicular to the axial plane at the anterior and posterior extent of the corpus callosum. Finally, a third coronal plane was created at the midpoint between the first two coronal planes, which divided the brain into anterior and posterior halves in each hemisphere. A detailed description can be found in our previous study [2].

### Statistical Analysis

Bivariate association between bipolar cases and controls and continuous variables (e.g. age) were conducted using t-tests, while their association with categorical variables were conducted using chi-squared tests. General linear model (GLM) was used to assess the associations between volumetric variables with bipolar status and BDNF genotype. These models were adjusted for age, gender, cerebrum volume and age of onset. These models also included two and three way interactions of bipolar status, BDNF genotype, age and sex. As a first step, whole brain grey and white matter volumes and CSF was evaluated before regional variations were explored. Similar analysis was conducted for BDNF Val66Met genotype, i.e. Met66 allele carriers and Val66 homozygotes. Significance of associations were assessed at alpha  = 0.05. All analyses were conducted using SAS 9.2 statistical software.

## Results

### Sample Characteristics

A total of 230 subjects were included in this study. 166 had a diagnosis of bipolar disorder, and 64 were healthy control subjects. In our study population, 92 subjects were BDNF Met66 allele carriers while the other 138 subjects were Val66 homozygotes. We found no deviation from Hardy-Weinberg equilibrium expectations. Due to the small number of Met66 homozygous individuals, Met66 carriers were grouped together and subsequently compared with Val66 homozygotes. There was no significant difference in gender between either population (bipolar vs. controls, and BDNF Met66 carriers vs. Val66 homozygotes) on univariate analysis, but difference in age of bipolar vs. control subjects was slightly significant at p = 0.0343. 46 of these subjects were from an earlier study we conducted [2], 34 of which were bipolar and the rest (12) were controls. Overall however, this study used a younger population with mean age 44.4 years for bipolar and 49.4 years for controls, compared to 60.5 years and 58.1 years respectively in the previous study. All subjects were of Caucasian race.

### Bipolar Status and Brain Volumes

Overall there were significant differences in total cerebrospinal fluid (CSF) and total grey matter between bipolar subjects and healthy controls. Differences were found in 38 variables in the first model associating bipolar status with brain volume. 26 of these variables had greater volumes in the bipolar population; these were all CSF variables (Table 2). Specifically, the CSF variables having a larger volume in bipolar subjects were: total CSF (including left and right total), total non ventricular CSF ( including total left, total right, anterior half non ventricular CSF (and including anterior quarter, right anterior half) and 7 parcellated regions), CSF in right and left anterior half (including left anterior quarter), right ventricular CSF, and total lateral ventricles (including parcellation regions 6, 8 and 9, lateral ventricles in right anterior half).

**Table 2:** Variables with larger volumes (ml) in bipolar subjects.

	Bipolar (n = 166)		Controls (n = 64)		
Brain Region	Mean	SD	Mean	SD	P-value
Total CSF	251.66	85.14	239.79	75.35	0.0121
Left total CSF	114.06	41.43	107.14	34.85	0.0037
Right total CSF	111.94	38.91	106.32	35.40	0.0121
Non-ventricular CSF in anterior half (Sum of regions 1,2,3,4,5,6,7, and 8)	94.56	31.75	88.38	29.36	0.0025
Non-ventricular CSF in anterior quarter (Sum of regions 1,2,3, and 4)	34.65	12.55	31.94	11.88	0.0066
Non-ventricular CSF in right anterior half	49.85	16.22	46.43	14.66	0.0024
CSF in right anterior quarter	18.79	6.53	17.06	5.89	0.0025
CSF in left anterior half	44.70	15.98	41.95	14.97	0.0042
CSF in left anterior quarter	15.86	6.30	14.87	6.18	0.025
Right ventricle	11.66	6.58	10.22	4.69	0.0099
Non-ventricular CSF	227.77	76.55	218.31	69.24	0.0217
Left non-ventricular CSF	101.83	36.82	95.89	31.69	0.0052
Right non-ventricular CSF	100.28	34.88	96.11	32.29	0.0274
Non-ventricular CSF in region 1	2.62	1.33	2.22	1.06	0.0098
Non-ventricular CSF in region 2	16.17	5.70	14.85	5.33	0.0064
Non-ventricular CSF in region 4	13.54	5.38	12.81	5.44	0.0398
Non-ventricular CSF in region 5	9.91	3.47	9.42	2.87	0.0481
Non-ventricular CSF in region 6	21.15	7.42	19.94	6.91	0.0065
Non-ventricular CSF in region 7	9.81	3.54	9.25	2.66	0.0241
Non-ventricular CSF in region 8	19.03	7.35	17.83	6.92	0.0026
Lateral ventricle in region 6	4.02	2.26	3.45	1.80	0.0045
Lateral ventricle in region 8	4.02	2.23	3.62	1.87	0.0308
Lateral ventricle in region 9	0.84	0.45	0.75	0.35	0.038
Lateral ventricle in right anterior half (Sum of regions 1,2,5, and 6)	4.16	2.28	3.59	1.87	0.0048
Total non-ventricular CSF in the cerebrum (Sum of regions 1–16)	201.99	71.23	191.96	63.52	0.0118
Total lateral ventricles (Sum of regions 1–16)	23.92	13.27	21.43	9.97	0.0234

The remaining 12 variables having greater volumes in the controls (Table 3) were all grey matter regions. These included total grey matter (including total left and right brain grey matter), total non lesion grey matter (cerebrum, left brain and right brain), non lesion grey matter in posterior half (including right posterior half) and parcellation regions 5, 7 and 15. Mean cerebrum volumes (and standard deviations) are as follows: bipolar 1163.66 (128.12) ml, healthy controls 1150.22 (120.20) ml. Three other brain regions – namely total white matter, left and right putamen are of particular interest to us because they were previously found to have significantly different volumes in bipolar versus healthy subjects [2], [3], [7]; however, they did not reach statistical significance in this study and are thus not reported here.

**Table 3:** Brain regions with larger volumes (ml) in healthy controls.

	Bipolar (n = 166)		Controls (n = 64)		
Brain Region	Mean	SD	Mean	SD	P-value
Total gray matter	637.22	91.30	641.14	106.73	0.0051
Left total gray matter	268.34	38.24	268.70	42.78	0.0221
Right total gray matter	270.80	39.49	271.69	45.06	0.0136
Non lesion gray matter	637.16	91.34	641.08	106.74	0.0051
Left non lesion gray matter	268.31	38.26	268.67	42.79	0.0216
Right non lesion gray matter	270.77	39.50	271.66	45.06	0.0136
Non lesion gray matter in region 5	31.98	5.39	32.71	4.91	0.0143
Non lesion gray matter in region 7	30.06	4.87	30.92	4.80	0.0042
Non lesion gray matter in region 15	21.83	6.16	23.73	6.50	0.0038
Non-lesion gray matter in posterior half (Sum of regions 9,10,11,12,13,14,15 and 16)	309.05	46.04	310.93	56.30	0.0197
Total non-lesion gray matter in the cerebrum	538.94	77.35	540.46	87.36	0.0144
Non-lesion gray matter in right posterior half (Sum of regions 9,10,13, and 14)	150.81	23.54	151.40	30.22	0.002

### BDNF Genotype and Brain Volumes

A second general linear model was run on the same population to examine brain morphological differences with BDNF Val66Met genotype as the independent variable, controlling for age, gender, cerebrum volume and bipolar status. We did not find a significant volumetric difference in total gray matter or total white matter between BDNF Val66 homozygotes and Met66 allele carriers.

Three-way interactions of bipolar status, BDNF genotype and age or sex were all non-significant.

## Discussion

Our study examined the effect of the BDNF Val66Met genotype on regional brain volumes in a cohort of 230 subjects, of which 166 were diagnosed with bipolar disorder and the rest (64) were healthy controls. To our knowledge, this is the first study examining the relationship of both bipolar status and BDNF Val66Met genotype on differences in regional brain volumes in a population of bipolar and healthy subjects. It expands our understanding of the influence of this genetic locus on brain morphometry, in the presence and absence of psychiatric illness.

### Bipolar Status and Brain Volumes

In our study, cerebrospinal fluid (CSF) regions, including total CSF, regional non ventricular CSF and the volume of lateral ventricles, in particular the right ventricle were increased in bipolar subjects compared to controls. One of the most pervasive neuroanatomical changes associated with bipolar disease is the enlargement of lateral and third ventricles [4], [39], a change similar to that seen in schizophrenia [40]. This is not unexpected given the overlap in clinical presentations of schizophrenia and bipolar disorder, such as the presence of psychotic features and disinhibition. In these patients, the enlarged ventricles are often at the expense of reduced gray and white matter volumes.

In line with our expectations, we detected a statistically significant difference in total and regional gray matter volumes. Specifically, we observed smaller gray matter volumes in parcellation regions 5, 7 and 15 in the bipolar subjects, corresponding to inferior frontal (regions 5 and 7) and inferior posterior (region 15) areas of the brain. These gray matter changes are consistent with structural abnormalities in bipolar disorder reported elsewhere [2], [41], suggesting that the postulated anterior limbic network of prefrontal-striatal-thalamic structures is likely to be responsible for mood-regulation. Additionally, when taken together with a previous study of ours [2] that used a population encompassing this study’s population but with an older mean age (mean age 60.5 and 58.1 years respectively in bipolar and healthy subjects, compared to 44.4 and 49.4 years in this study), it suggests that significant gray matter reductions in bipolar subjects are already present at a younger age and may be accelerated with aging.

We did not find an effect of bipolar status on white matter volumes in our study. The current body of evidence is mixed; several studies did not find a difference in white matter volumes similar to ours [2], [41], while a recent study on an Asian population [3] found loss of white matter networks involving frontal and prefrontal areas in bipolar subjects compared to healthy controls.

### BDNF Genotype and Brain Volumes

We did not find any differences in brain regions or specific structures as might be predicted by other studies [23], [30], in particular, gray matter volume [30] and dorsolateral prefrontal cortex [23]. A study by [42] reported a global effect of BDNF Val66Met polymorphism on brain volumes independent of age and sex, with Val66 homozygotes having significantly larger total lobar volumes, particularly in temporal and frontal lobe white matter. However, none of these differences were borne out in our study, perhaps a result of differences in patient populations, MRI acquisition and segmentation protocols. While we would have liked to look at the effect of BDNF Val66Met genotype on structures such as the amygdala and caudate nucleus previously reported in other studies [16], [24], insufficient numbers prevented us from doing so.

### Limitations of Study

There are several limitations to our study. First, we were unable to test for a dose-dependent effect of the BDNF Met66 allele on brain morphology; given the much lower frequency of the homozygous Met66 genotype, a larger sample would be needed. Second, as the hypotheses are exploratory, no adjustments were made for multiple testing. However, the reported number of tests conducted here is about 36. At alpha  = 0.05, Bonferroni adjusted alpha [43] is 0.05/36 = 0.0014. With this adjusted alpha the significant results presented here will no longer be significant.”

Finally, though we were able to control for age, gender and cerebrum volume, several other clinical characteristics of our sample population could influence volumetric brain matter changes. An example would be co-morbid conditions such as substance abuse and anxiety, which although part of our exclusion criteria, may not be easily picked up by clinicians at the point of enrollment into the study. More importantly, our study did not control for the effect of medication – we did not classify patients based on their medication status (whether they were on mood stabilizers or other psychiatric medications) and duration of treatment prior to study enrolment. This is a potential confounding factor, as increases in grey matter density have been found in lithium-treated bipolar patients [44] and following a 4-week trial of lithium in healthy volunteers [45]. Thus, the use of medications in our patient population may obfuscate the relationship between bipolar status, BDNF genotype and brain volumes.

### Conclusion

Overall, our observations of decreased total and regional gray matter volumes, particularly in inferior and posterior parts of the brain, together with a concomitant increase in cerebrospinal fluid in bipolar patients, suggest that these regions may play a role in both mood and cognitive symptoms of bipolar disorder, a condition that appears to be increasingly similar to schizophrenia in both clinical manifestations and neuroanatomical changes. The presence of a BDNF Met66 or Val66 allele (BDNF Val66Met polymorphism) does not appear to confer an effect on regional brain volumes in either healthy or bipolar patients.

Additional studies enabling us to correlate such findings with subjects' clinical course of bipolar disorder (age of onset, family history and severity of both depressive and manic symptoms, etc.) may shed more light on the impact of disease on volumetric regional brain changes. It would also be instructive to further split the study population into four subgroups along both dimensions (genotype and bipolar status), so as to examine the effect of BDNF Val66Met genotype on bipolar and healthy subjects separately.
